# Gelation upon the Mixing of Amphiphilic Graft and Triblock Copolymers Containing Enantiomeric Polylactide Segments through Stereocomplex Formation

**DOI:** 10.3390/gels10020139

**Published:** 2024-02-09

**Authors:** Yuichi Ohya, Yasuyuki Yoshida, Taiki Kumagae, Akinori Kuzuya

**Affiliations:** 1 Department of Chemistry and Materials Engineering, Faculty of Chemistry, Materials and Bioengineering, Kansai University, 3-3-35 Yamate, Suita 564-8680, Osaka, Japankuzuya@kansai-u.ac.jp (A.K.); 2 Kansai University Medical Polymer Research Center (KUMP-RC), Organization for Research and Development of Innovative Science and Technology (ORDIST), Kansai University, 3-3-35 Yamate, Suita 564-8680, Osaka, Japan

**Keywords:** hydrogel, injectable polymers, stereocomplex, polylactide, graft copolymers

## Abstract

Biodegradable injectable polymer (IP) systems that form hydrogels in situ when injected into the body have considerable potential as medical materials. In this paper, we report a new two-solution mixed biodegradable IP system that utilizes the stereocomplex (SC) formation of poly(l-lactide) (PLLA) and poly(d-lactide) (PDLA). We synthesized triblock copolymers of PLLA and poly(ethylene glycol), PLLA-*b*-PEG-*b*-PLLA (*tri*-L), and a graft copolymer of dextran (Dex) attached to a PDLA-*b*-PEG diblock copolymer, Dex-g-(PDLA-*b*-PEG) (*gb*-D). We found that a hydrogel can be obtained by mixing *gb*-D solution and *tri*-L solution via SC formation. Although it is already known that graft copolymers attached to enantiomeric PLLA and PDLA chains can form an SC hydrogel upon mixing, we revealed that hydrogels can also be formed by a combination of graft and triblock copolymers. In this system (graft vs. triblock), the gelation time was shorter, within 1 min, and the physical strength of the resulting hydrogel (G′ > 100 Pa) was higher than when graft copolymers were mixed. Triblock copolymers form micelles (16 nm in diameter) in aqueous solutions and hydrophobic drugs can be easily encapsulated in micelles. In contrast, graft copolymers have the advantage that their molecular weight can be set high, contributing to improved mechanical strength of the obtained hydrogel. Various biologically active polymers can be used as the main chains of graft copolymers, and chemical modification using the remaining functional side chain groups is also easy. Therefore, the developed mixing system with a graft vs. triblock combination can be applied to medical materials as a highly convenient, physically cross-linked IP system.

## 1. Introduction

Some polymer solutions exhibit sol-to-gel transitions in response to external stimuli or the mixing of two solutions; such polymer solutions can be used as injectable polymer (IP) systems for medical applications [1,2,3,4,5]. The polymer solution can be easily injected into the body by a syringe or catheter and eventually becomes a hydrogel at the deposition site in the body. Living cells [6,7,8,9] and drugs [10,11,12] (e.g., bioactive reagents, proteins, peptides, and nucleic acids) can be mixed with polymer solutions in the sol state. After administration, the solution mixture can form a hydrogel, entrapping the cells or drugs to the administered site of the body without a surgical approach. Particularly, IP systems that use biodegradable or bioabsorbable polymers can provide minimally invasive methods for delivering implant materials for medical use. Moreover, such IP hydrogels are expected to be used as barriers to prevent postoperative peritoneal adhesion [13,14,15] and endoscopic submucosal dissection (ESD) [16], and as embolization agents for therapy [17,18,19].

IP hydrogels consist of a three-dimensional network of water-soluble (hydrophilic) polymers connected via chemical or physical cross-linking. Physically cross-linked hydrogels can be formed in response to environmental stimuli such as temperature increases and pH changes [20,21,22]. Temperature-responsive polymer solutions exhibit rapid gelation in response to temperature increases up to body temperature upon injection. However, the mechanical strength of hydrogels is often not sufficiently high because of the instability of physical cross-linking.

By contrast, the chemically cross-linked IP hydrogel can form a hydrogel upon mixing two polymer solutions, which react with each other, or polymers and cross-link-forming reagents (cross-linkers or enzymes) [23,24,25,26,27,28,29,30]. Two-solution mixing systems using Schiff base formation, Michael addition, succinimide–amine coupling, and click chemistry (thiol-ene, alkyne-azide, and diels-alder) reactions have been reported [27,28,29,30]. The chemically cross-linked hydrogels showed relatively higher mechanical strength than those physically cross-linked (non-covalently). However, precise control of the gelation time is difficult. Polymers bearing polymerizable (typically vinyl) groups have also been reported as in situ gelling systems triggered by external stimuli (typically ultraviolet (UV) light irradiation) [24]. However, the polymer or oligomer chains produced during polymerization are not biodegradable, and the cross-linking reagents and UV irradiation are potentially toxic.

Poly(l-lactide) (PLLA) is a well-known biodegradable polymer used in biomedical applications. An equivalent mixture of PLLA and its enantiomer poly(d-lactide) (PDLA) can form a stable stereocomplex (SC) crystal [31,32,33]. The SC crystal has a higher melting point (approximately 230 °C) than the homo crystal (approximately 180 °C). While pure PLLA and PDLA crystallize in an orthorhombic form with a 10/3 helix [34] in their conformation, the stereocomplex has a triclinic form with a 3/1 helix to maximize the van der Waals interactions between adjacent helices with opposite chirality [35]. Consequently, the mechanical strength of the polylactide material containing SC crystals was higher than that of the material containing only homo crystals. Hennink et al. reported an in situ gelling system through the SC formation of graft copolymers using dextran (Dex) with enantiomeric graft chains, Dex-*g*-PLLA, and Dex-*g*-PDLA [36,37,38]. These graft copolymers formed a hydrogel upon mixing with polymer solutions in the absence of a cross-linking agent. SC-cross-linked hydrogel systems may have an advantage in terms of stability compared to physical hydrogels formed by simple hydrophobic interactions. Kimura et al. also utilized SC formation as a driving force for hydrogel formation between ABA triblock copolymers of PLLA or PDLA and poly(ethylene glycol) (PEG), PLLA-*b*-PEG-*b*-PLLA, and PDLA-*b*-PEG-*b*-PDLA triblock copolymers [39].

**Figure 1:**
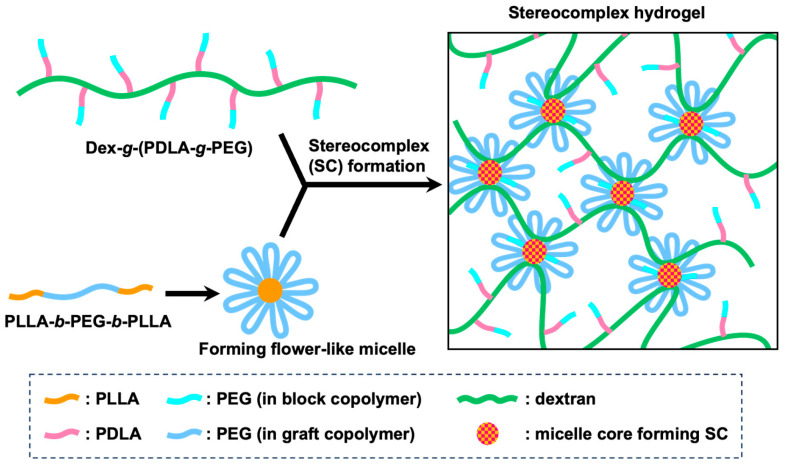
Schematic illustration of the polymers, Dex-*g*-(PLLA-*b*-PEG) (*gb-*L), Dex-*g*-(PDLA-*b*-PEG) (*gb-*D), and PLLA-*b*-PEG-*b*-PLLA (*tri*-L) used in this study and hydrogel formation by mixing of *gb-*D and *tri-*L.

In the aforementioned SC gelation systems [36,37,38,39], polymer combinations with the same architecture (graft or block) of the same hydrophilic polymer (Dex or PEG) attached to the PLLA or PDLA segments were mixed to form a hydrogel: Dex-graft vs. Dex-graft or PEG-triblock vs. PEG-triblock. They were “symmetric” combinations. Here, we propose the simple question: must they have the same architecture and hydrophilic segments? In this study, we investigated the possibility of SC hydrogel formation by mixing polymers with different architectures: graft versus triblock (Figure 1). Dex-*g*-PDLA and Dex-*g*-PLLA were synthesized in the preliminary stages of this study. However, some graft copolymers with a relatively high degree of PL(D)LA chain introduction are poorly water-soluble. Therefore, we attached a short methoxy-PEG (MeO-PEG) chain extension to the PDLA (or PLLA) graft chains to maintain water solubility and obtained Dex-*g*-(PDLA-*b*-PEG) (*gb*-D) and Dex-*g*-(PLLA-*b*-PEG) (*gb*-L). Using this polymer architecture, it was possible to introduce a relatively large number of PDLA (or PLLA) chains per Dex molecule. We synthesized triblock copolymers of PLLA and PEG (PLLA-*b*-PEG-*b*-PLLA, *tri*-L) and investigated hydrogel formation upon mixing the block copolymer with the graft copolymer. We found that the SC hydrogel could be obtained by mixing the graft (*gb*-D) and triblock (*tri*-L).

The combination of graft- and triblock-forming SC hydrogels has several advantages. The synthesis of graft copolymers is relatively complicated compared with that of triblock copolymers, and PLLA-*b*-PEG-*b*-PLLA is much easier to synthesize. A triblock copolymer with one enantiomer (e.g., PLLA) can be used as a “cross-linker” for a counter graft copolymer with another enantiomer (e.g., PDLA) to form an SC hydrogel; we do not need to synthesize both enantiomeric graft copolymers. Moreover, triblock copolymers (e.g., PLLA-*b*-PEG-*b*-PLLA) dissolve in water, forming flower-like micelles at concentrations above the critical micelle concentration (CMC) (Figure 1). Incorporating hydrophobic substances (typically drugs) into micelles facilitates their entrapment of the hydrophobic substances in the SC hydrogel. One of the critical factors affecting the physical properties of the obtained hydrogels is the molecular weight of the component polymers; a higher molecular weight leads to higher mechanical strength. However, it is difficult to increase the molecular weight of the polymer components in a mixing system using triblock copolymers. It is much easier to provide a graft copolymer with a higher molecular weight, which can help increase the physical strength of the obtained SC hydrogel. Moreover, various hydrophilic polymers can be used as the main chains of graft copolymers, such as biologically active or enzymatically degradable polysaccharides (e.g., hyaluronic acid and heparin). The reactive side chain groups in the main chain can be used for further chemical modifications with drugs, fluorescent dyes, or specific ligands for biological (cellular) recognition. So, this combination to form SC hydrogels must increase the usefulness of the SC hydrogel system as medical materials.

## 2. Results and Discussion

### 2.1. Synthesis of the Copolymers

**Scheme 1:**
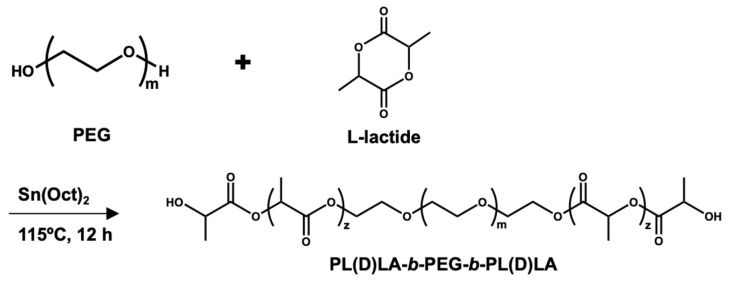
Synthesis of PLLA-*b*-PEG-*b*-PLLA (*tri*-L).

**Scheme 2:**
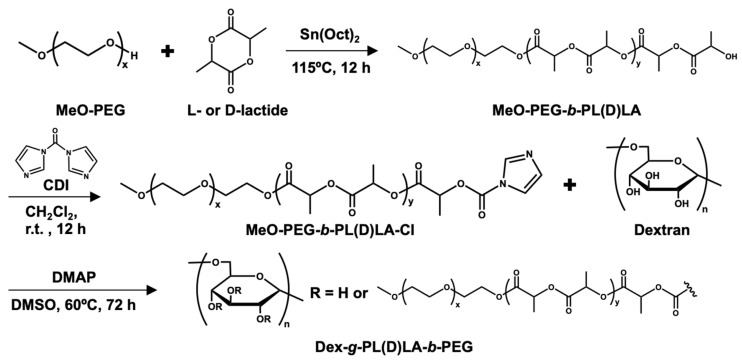
Synthesis of Dex-*g*-PLLA-*b*-PEG and Dex-*g*-PDLA-*b*-PEG.

**Figure 2:**
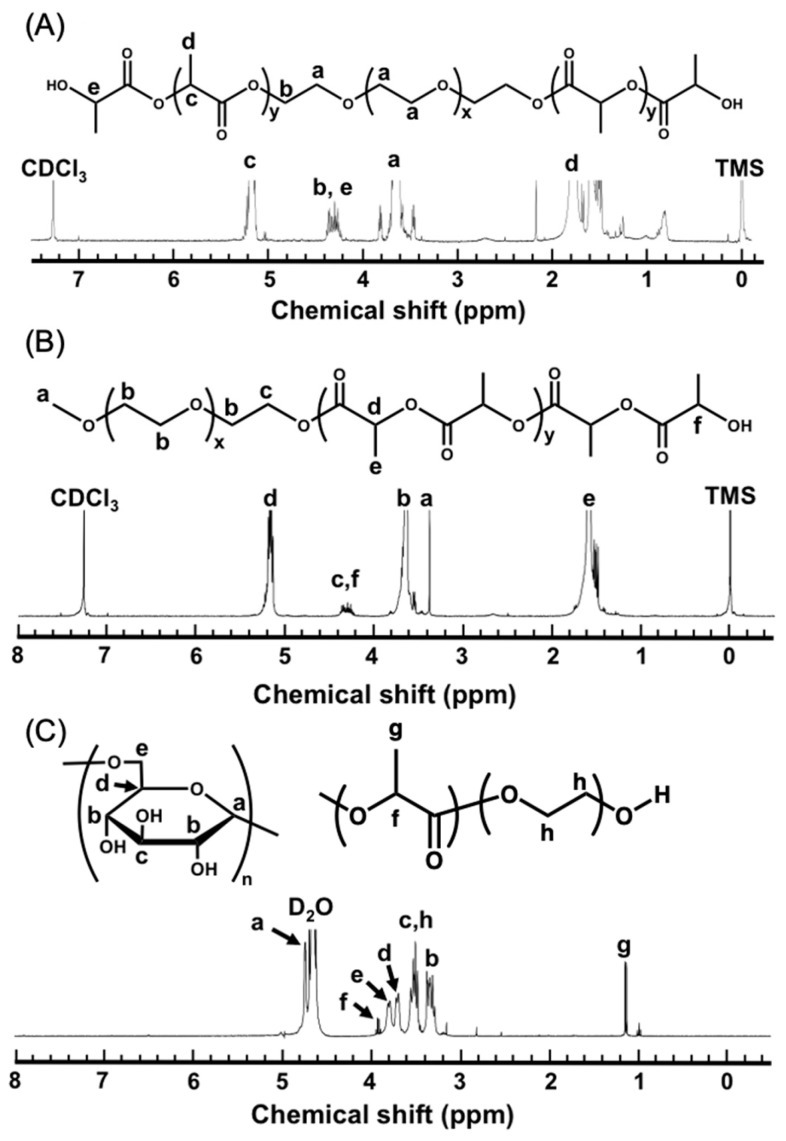
^1^H-NMR spectra of (**A**) PLLA-*b*-PEG-*b*-PLLA triblock copolymer (*tri*-L) (solvent: CDCl_3_), (**B**) MeO-PEG-*b*-PDLA diblock copolymer (*b*-D) (solvent: CDCl_3_), and (**C**) Dex-*g*-(PDLA-*b*-PEG) (*gb*-D) (solvent: D_2_O/NaOD). The lowercase letters a–d for (**A**), a–f for (**B**) and a–h for (**C**) indicate the assignments of each peak.

Block and graft copolymers were synthesized according to Scheme 1 and Scheme 2, respectively. Diblock copolymers, MeO-PEG-*b*-PLLA (*b*-L) and MeO-PEG-*b*-PDLA (*b*-D), were obtained by the ring-opening polymerization of L-Lactide and D-Lactide using MeO-PEG (molecular weight (MW) = 1000 g/mol) as a macroinitiator. The triblock copolymer (PLLA-*b*-PEG-*b*-PLLA) was synthesized using the same method using PEG (MW = 4600 g/mol) as a macroinitiator to produce *tri*-L. The characteristics of *b*-L, *b*-D, and *tri*-L are summarized in Table 1. The H-NMR spectra of *tri*-L and *b*-D are shown in Figure 2A,B. The MWs of the PLLA and PDLA segments in *b*-L and *b*-D are 2900 or 2800 g/mol, respectively. The molecular weight (MW) of one PLLA segment in *tri*-L is 1200 g/mol. The obtained *b*-L and *b*-D showed sticky paste morphologies, while *tri*-L showed a powdery solid morphology at room temperature in the dry state. Graft copolymers, Dexs attached to MeO-PEG-*b*-PLLA or MeO-PEG-*b*-PDLA graft chains (*gb*-L or *gb*-D), were obtained by coupling dextran (MW = 150,000) with *b*-L or *b*-D. The characteristics of the obtained graft copolymers are summarized in Table 2. Typical ^1^H nuclear magnetic resonance (NMR) spectra of the copolymers are shown in Figure 2C. The degree of polymerization of each PL(D)LA chain in the copolymers and the number of graft chains (*b*-L or *b*-D) attached per Dex molecule were calculated from the ^1^H-NMR spectra. The number of graft chains per Dex molecule for *gb*-D were approximately four. The obtained *gb*-D showed a cotton-like solid morphology in the dry state at room temperature after freeze-drying.

### 2.2. Gelation Behavior of the Mixture Solution

**Figure 3:**
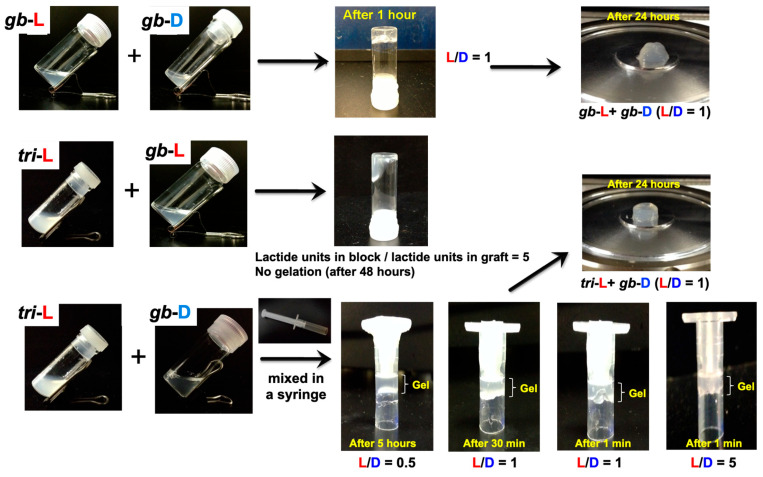
Photographs of the typical examples of polymer solutions before mixing and after gelation. The final polymer concentrations are shown in Table 3.

The gelation behavior of the mixture solution of *gb*-D and *tri*-L or *gb*-L was investigated by the test-tube inversion method [6] at 37 °C. The total graft copolymer concentration was adjusted to 8.0 wt%. The results for various combinations and mixing ratios are summarized in Table 3. Photographs of typical combinations are shown in Figure 3. The mixture of *gb*-D (3.6 wt%) and *gb*-L (4.4 wt%) (total graft copolymer concentration = 8.0 wt% and L-lactide units (mol)/D-lactide unit (mol) (L/D) = 1.0) showed gelation in 51 min after mixing. The storage modulus of the hydrogel obtained was 5.0 Pa. A mixture of two graft copolymers with opposite enantiomeric polylactide segments showed gelation upon mixing, a result similar to that obtained in a previous report [33]. The introduction of a short PEG chain at the terminus of the graft PD(L)LA chains did not interfere with SC formation in the graft copolymers.

**Figure 4:**
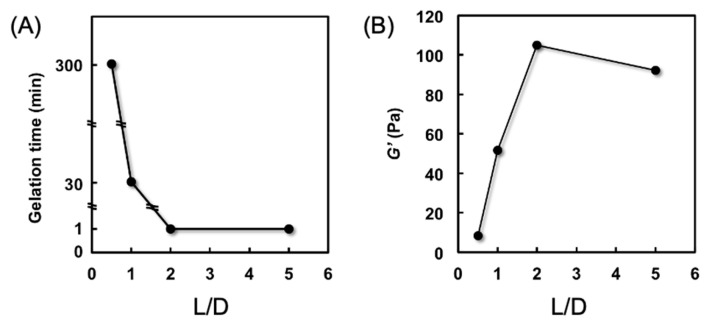
Effects of L-lactide unit/D-lactide unit ratio (L/D) on (**A**) gelation time and (**B**) storage modulus (G′) 24 h after mixing at 37 °C for the combination of PLLA-*b*-PEG-*b*-PLLA (*tri*-L) and Dex-*g*-(PDLA-*b*-PEG) (*gb-*D).

We investigated the combination of a graft/triblock with opposite enantiomeric polylactide chains, *tri*-L and *gb*-D. The gelation times and storage moduli of the mixed solutions as functions of L/D are plotted in Figure 4. When L/D was 0.5 and 1.0, the gelation times of the mixture solutions were 300 and 30 min, and the storage moduli were 8.4 and 52 Pa, respectively. Gelation can be induced by mixing the triblock and graft copolymers. Moreover, rapid gelation was observed when the L/D was 2 and 5; the gelation time was shorter than 1 min. The storage moduli of these systems were 105 and 92 Pa. These results indicate that the larger the L/D ratio, the shorter the gelation time and the higher the storage modulus of the resulting hydrogel. A mixture of *tri*-L and *gb*-L, with the same L-form enantiomeric chain, did not form a hydrogel 48 h after mixing. These results suggest that gelation of the mixture of *tri*-L and *gb*-D was induced by SC formation between the PLLA segments in *tri*-L and the PDLA segments in *gb*-D.

*Tri*-L contains two PLLA chains (MW = 1200 × 2 = 2400 g/mol). One micelle contains several to several dozen block copolymers; if we consider that the aggregation number of a micelle is approximately 10, the total number of PLLA segments in a micelle is 24,000 g/mol. The PDLA segment in the graft chain of *gb*-D was 2800 g/mol, and five graft chains were introduced per dextran molecule, which is 5.3 (Table 2). Therefore, the total MW of the PDLA segments in one *gb*-D molecule was 2800 × 5.3 = 14,840 g/mol. If one micelle has 24,000 (g/mol) PLLA chains, when L/D = 2.0, 1.0, and 0.5, the ratios of *N*_m_ (number of micelles)/*N*_gp_ (number of graft copolymers) = 3.2, 1.6, and 0.8, respectively (48,000/14,840, 24,000/14,840, and 12,000/14,840). For simplicity, assuming that two graft chains are inserted into one micelle, the molecular weight of the polymer aggregate can theoretically become infinite (gelling) with at least *N*_m_/*N*_gp_ = 1; a percolation transition for gelation can occur when *N*_m_/*N*_gp_ is larger than 1. When L/D = 0.5, *N*_m_/*N*_gp_ = 0.81 < 1. Therefore, this calculation suggests when L/D = 0.5, three (or more) graft chains must be inserted into one micelle. It is reasonable that the insertion of a second graft chain into a micelle is slower and less frequent than the insertion of the first chain into the same micelle and that the insertion of a third graft chain into a micelle is much slower and less frequent than the insertion of the second chain into the same micelle because of spatial constraints and steric hindrance. The drastic change in the gelation time between L/D = 1 and 2 (*N*_m_/*N*_gp_ = 3.2 and 1.6) may reflect the stoichiometry discussed above and differences in the insertion rates of the first, second, and third graft chains into the same micelle.

Interestingly, the combination of *tri*-L/*gb*-D(1) (L/D = 1.0) showed a shorter gelation time and a higher storage modulus than the combination of graft copolymers (*gb*-L/*gb*-D) with the same L/D ratio of 1.0. Moreover, the mixtures with L/D more than 2.0 showed significantly shorter gelation times and higher storage moduli. Shorter gelation times and higher storage moduli are favorable for IP systems. A hydrogel with a shorter gelation time can prevent flow from the injection site during administration. Higher storage moduli may provide higher physical stability and a longer duration for the hydrogels in the body. Therefore, the triblock and graft copolymer mixing system developed in this study can provide a more favorable IP system through accessible synthetic procedures.

**Figure 5:**
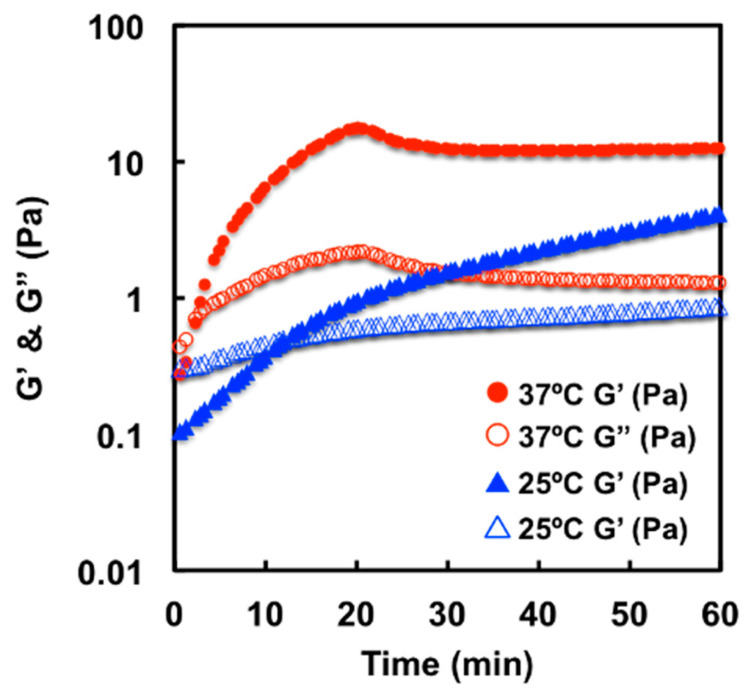
Time course of storage modulus (G′, closed symbols) and loss modulus (G″, open symbols) for the mixture of PLLA-*b*-PEG-*b*-PLLA (*tri*-L) and Dex-*g*-(PDLA-*b*-PEG) (*gb-D*) (L/D = 2.0) at 37 °C (red circles) and 25 °C (blue triangles).

Figure 5 shows the time-course dynamic rheological measurements after mixing *tri*-L and *gb*-D (L/D = 2.0) at 37 and 25 °C. When G′ takes over G″, it is recognized as gelation time. Naturally, the gelation time depends on the temperature: the higher the temperature, the more rapid the gelation. The mixture of *tri*-L and *gb*-D showed rapid gelation (within 1 min) at body temperature (37 °C); however, at 25 °C, around room temperature (r.t.), gelation took 13 min. The results indicate that the mixture did not show gelation for 13 min after mixing at room temperature and formed a hydrogel rapidly upon injection by heating to body temperature. Such gelation behavior must be favorable and convenient at the clinical stage to provide a medical doctor with more time for handling after mixing before injection.

**Figure 6:**
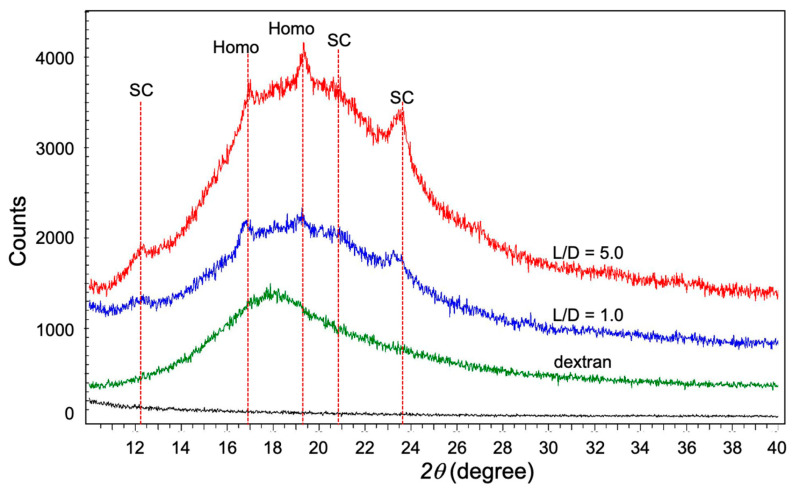
Wide-angle X-ray diffraction (WAXD) spectra for the mixture of *tri-*L and *gb-*D (L/D = 1.0 and 5.0) after freeze-drying and intact dextran.

We obtained wide-angle X-ray diffraction (WAXD) patterns to confirm SC formation in the hydrogels. Figure 6 shows typical WAXD patterns for the *tri*-L and *gb*-D mixture hydrogels with L/D = 1.0 or 5.0 after freeze-drying, and intact Dex as typical examples. The PLLA and PDLA SC crystals showed specific diffraction peak angles (2θ) of approximately 12, 21, and 24° [40,41]. The homo PL(D)LA crystals show peaks at approximately 2θ = 17 and 19°, respectively. The mixtures of *tri*-L and *gb*-D (L/D = 1.0 and 5.0) showed clear differentiation peaks around 2θ = 12, 17, 19, and 24° and weak peaks around 21°. Diffraction peaks were observed for both homo and SC crystals. The mixture with L/D = 5.0, which exhibited rapid gelation and a higher storage modulus, showed a higher intensity of these peaks than that with L/D = 1/0. These results suggest that SC complex crystals formed for both mixtures (L/D = 1.0, 5.0); however, the crystal formation efficiency of L/D = 5.0 was higher than that of L/D = 1.0. These results suggest that the rapid hydrogel formation and higher storage modulus of the mixture (L/D = 5.0) are due to its higher SC formation.

**Figure 7:**
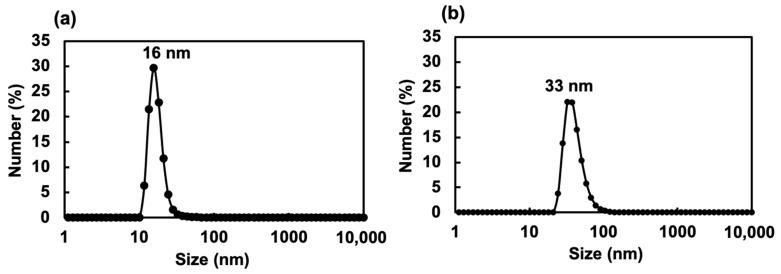
Results of dynamic light scattering (DLS) measurement for *tri*-L solution (**a**) and *gb*-D so lution (**b**) in water at 25 °C expressed as number distribution. Polymer concentration = 0.2 wt%.

The triblock copolymer dissolves by the formation of flower-like micelles in water. We also performed dynamic light scattering (DLS) measurements for *tri-*L and *gb-*D (Figure 7). The average diameter of *tri-*L in water was 16 nm. These results support the micelle formation of *tri-*L in aqueous solutions. Considering the above results, a possible gelation mechanism for the mixture of *tri-*L and *gb-*D can be proposed, as illustrated in Figure 1. Upon mixing, the side chains of the graft copolymer (*gb-*D) containing PDLA are inserted into the *tri-*L micelle cores to form stable SC crystals. The *tri-*L micelles connect two or more graft chains and can act as cross-linking points in the hydrogel. Each micelle in the gel state must contain PLLA chains that do not participate in the SC crystal. Therefore, an excess amount of PLLA in *tri-*L may be required to form a stable hydrogel in a short time.

## 3. Conclusions

In this study, we synthesized a triblock copolymer, PLLA-*b*-PEG-*b*-PLLA (*tri*-L), and a graft copolymer, Dex-*g*-(PDLA-*b*-PEG) (*gb*-D). We then obtained a biodegradable physically cross-linked SC hydrogel by mixing *tri*-L aqueous and *gb*-D aqueous solutions. We demonstrated that SC hydrogels could be formed even when the polymers did not have the same architecture (graft/graft or triblock/triblock copolymers). There are several reports on two-solution mixed physical gelation systems. However, to the best of our knowledge, there have been no reports of a two-solution-mixing physical gelation system using graft copolymers and block copolymers. Physical hydrogels can be obtained by mixing graft and triblock copolymers. Therefore, triblock copolymers with PLLA chains (*tri-*L in this case) can act as a “cross-linker” for various graft copolymers containing PDLA graft chains. We found that SC formation occurred more efficiently for the triblock with a graft combination than between grafts; hydrogels with higher mechanical strength could be obtained in a shorter gelation time.

Additionally, gelation systems using graft/triblock combinations have several advantages over mixing with graft/graft or triblock/triblock combinations. Hydrophobic–hydrophilic–hydrophobic ABA-type triblock copolymers form micelles and dissolve in water above the CMC. Using micelles that entrap hydrophobic substances (such as drugs) in their core, hydrogels containing these substances can be easily prepared. Furthermore, for graft copolymers, the main chain of the graft copolymers with desired properties and molecular weights can be chosen from a wide variety of water-soluble polymers. The mechanical strength of a physically cross-linked hydrogel depends on the molecular weight of the polymer used; thus, the strength can be expected to increase as the molecular weight increases. In addition, biologically active polysaccharides (such as heparin or hyaluronic acid) can be used as main chains to obtain biologically functional hydrogels. For example, when a polysaccharide that is selectively decomposed by a specific enzyme is used in the main chain, a hydrogel showing specific enzyme degradation can be obtained. Unreacted side chain functional groups are expected to remain in the graft polymer, and these residual functional groups can be easily functionalized by attaching cellular-recognizable peptides, drugs, fluorescent dyes, and others. This study provides a simpler and more versatile injectable hydrogel system based on SC formation. The biodegradable two-solution-mixed SC gelation systems developed in this study are expected to be applied as sustained drug-releasing systems, antiadhesion materials for surgical operations, cellular delivery systems, and scaffolds for tissue regeneration.

## 4. Materials and Methods

### 4.1. Materials

L-Lactide and D-lactide were obtained from Musashino Chemical Laboratory, Ltd. (Tokyo, Japan). PEG (number-averaged molecular weight (*M_n_*) = 4600) and monomethoxy-PEG (*M_n_* = 1000) were purchased from Sigma-Aldrich (St. Louis, MO, USA). Carbonyl diimidazole (CDI), tin-2-ethylhexanoate, and dextran (Dex) (*M_n_* = 150,000) were obtained from Fujifilm Wako Pure Chemical Co. (Osaka, Japan). All other chemicals were of reagent grade and were used without further purification.

### 4.2. Synthesis of PLLA-b-PEG-b-PLLA Triblock Copolymers

The synthesis of PLLA-*b*-PEG-*b*-PLLA triblock copolymers, *tri*-L and *tri*-D, was performed by ring-opening polymerization of L-Lactide or D-Lactide in the presence of PEG as a macroinitiator and tin-2-ethyl hexanoate as a catalyst in bulk at 115 °C for 12 h (Scheme 1). After polymerization, the products were purified by reprecipitation using chloroform as a good solvent and diethyl ether as a poor solvent. The reaction products were dissolved in chloroform, and the copolymer solutions were dropped onto ice-cooled diethyl ether. The white precipitate obtained was dried under a vacuum for 24 h. The *M_n_* and polydispersity index (*M_w_/M_n_*) of the obtained copolymers were characterized by ^1^H nuclear magnetic resonance (^1^H-NMR) spectroscopy using a JNM-GSX-400 (JEOL, Tokyo, Japan, solvent: CDCl_3_) and size-exclusion chromatography (SEC) (column: Toso TSKgel Multipore HXLM × 2, detector: RI, eluent: DMF, standard: PEG, and flow rate = 1.0 mL/min at 40 °C).

### 4.3. Synthesis of Dex-g-(PLLA-b-PEG) and Dex-g-(PDLA-b-PEG)

The synthesis of graft copolymers, Dex-*g*-(PLLA-*b*-PEG) (*gb*-L) and Dex-*g*-(PDLA-*b*-PEG) (*gb*-D), was carried out according to Scheme 2. MeO-PEG-*b*-PLLA (*b*-L) and MeO-PEG-*b*-PDLA (*b*-D) diblock copolymers were synthesized by the same method as described above for the triblock copolymers, using MeO-PEG as a macroinitiator instead of PEG (*M_n_* = 4600). The terminal hydroxyl groups of *b*-L or *b*-D diblock copolymers were converted into active carbonyl imidazole groups using CDI to produce *b*-L-CI or *b*-D-CI. The *b*-L or *b*-D diblock copolymers and CDI were dissolved in dry dichloromethane (DCM) and stirred for 12 h at 25 °C under an argon atmosphere. The reaction mixture was purified by reprecipitation using DCM and a mixture of *n*-hexane and ethanol (7:3) as the solvents. The white precipitate obtained was dried under a vacuum for 24 h. The *b*-L-CI, *b*-D-CI, and Dex were separated and dissolved in dimethyl sulfoxide (DMSO). The solution of *b*-L-CI or *b*-D-CI was added to the Dex solution and stirred for 72 h at 60 °C. The products were purified by reprecipitation and dialysis to remove unreacted *b*-L or *b*-D. The mixture solution was dissolved in DMSO and added dropwise into a large amount of acetone at 25 °C. The resulting precipitate was filtered and dissolved in DMSO. The obtained solution was transferred into a dialysis tube (molecular weight cut-off: MWCO = 3500) and dialyzed against ultrapure water for 48 h to remove DMSO. The aqueous solution obtained was freeze-dried. The *gb*-L and *gb*-D samples exhibited cotton-like solid morphologies at room temperature in the dry state. The degrees of introduction of *b*-L or *b*-D chains per Dex molecule and molecular weights for *gb*-L and *gb*-D were estimated by ^1^H-NMR (solvent: NaOD/D_2_O) and SEC (column: Toso TSKgel Multipore HXLM ×2, detector: RI, eluent: DMSO, standard: polystyrene, and flow rate = 0.3 mL/min at 40 °C).

### 4.4. DLS Measurement of Copolymer Solutions

The hydrodynamic diameters for *tri*-L and *gb*-D aggregates in aqueous solution were measured using dynamic light scattering (DLS) (Zetasizer nano Z ZEN2600, Malvern Instruments Ltd., Malvern, UK) at 25 or 37 °C at a detection angle of 173° with a He–Ne laser as the incident beam. The *tri*-L and *gb*-D were dissolved in water (0.2 wt%) and were filtered using a Millex (Millipore, Burlington, MA, USA) 0.8 μm membrane before measurement.

### 4.5. Gelation Behavior

The obtained copolymers were separately dissolved in water and then mixed in the combinations and concentrations as described in Table 3. Gelation of the polymer mixture solution was investigated using the test-tube inversion method [6]. Each copolymer solution was prepared at a given concentration by stirring overnight in water. Solutions containing each polymer were mixed at room temperature in a test tube or syringe with a tip cut-off.

### 4.6. Mechanical Strength of the Hydrogel

Rheological measurements were carried out using a dynamic rheometer (Thermo HAAKE RS600, Thermo Fisher Scientific, Waltham, MA, USA). A solvent trap was used to prevent solvent vaporization. The diameter of the parallel plate was 25 mm, and the gap was 1.0 mm. The controlled stress and frequency were 4.0 dyn/cm^2^ and 1.0 rad/s, respectively. To measure the storage modulus of the hydrogels after 24 h, the hydrogel formed in a syringe was kept at 25 °C for 24 h with sealing, then pushed out from the syringe, and the storage modulus was measured in compressive mode.

### 4.7. Wide-Angle X-ray Diffraction Analysis

The wide-angle X-ray diffraction (WAXD) patterns of the lyophilized hydrogel were obtained using an M18XHF22-SRA instrument (Bruker Japan, former MAC Science Co., Kanagawa, Japan) with Cu KR source (*λ*) 1.54 (Å) at 25 °C.

## Figures and Tables

**Table 1:** Characterization of MeO-PEG-*b*-PL(D)LA diblock and PLLA-*b*-PEG-*b*-PLLA triblock copolymers.

	Code Name	*M_n_* of PEG (g/mol)	*M_n_* of PL (D) LA (g/mol) ^1^	DP of PL(D)LA ^1^	*M_w_/M_n_* ^2^
MeO-PEG-*b*-PDLA	*b*-D	1000	2800	19	1.09
MeO-PEG-*b*-PLLA	*b*-L	1000	2900	20	1.11
PLLA-*b*-PEG-*b*-PLLA	*tri*-L	4600	1200	8.1	1.07

^1^ *M_n_* of the PL(D)LA segments and the degree of polymerization of the lactide unit were estimated by ^1^H-NMR (solvent: CDCl_3_). ^2^ Determined by size-exclusion chromatography (eluent: DMF, standard: PEG).

**Table 2:** Characterization of Dex-*g*-(PDLA-*b*-PEG) and Dex-*g*-(PLLA-*b*-PEG) copolymers.

	Code Name	*M_n_* × 10^−4^ (g/mol) ^1^	*M_w_/M_n_* ^2^	No. of Side Chains ^3^
Dex-*g*-(PLLA-*b*-PEG)	*gb*-L	16.6	2.80	4.0
Dex-*g*-(PDLA-*b*-PEG)	*gb*-D	17.0	2.82	5.3

^1^ Calculated from the results of ^1^H-NMR (solvent: CDCl_3_). ^2^ Determined by size-exclusion chromatography (eluent: dimethyl sulfoxide (DMSO), standard: pullulan). ^3^ Average number of side chains estimated by ^1^H-NMR (solvent: NaOD/D_2_O).

**Table 3:** Results of gelation behavior upon mixing of the polymers ^1^.

Sample	*tri*-L (wt%)	*gb*-D (wt%)	*gb*-L (wt%)	L/D (Block/Graft) ^2^	Sol or Gel	Gelation Time (min)	Storage Modulus after 24 h (Pa)
***gb*-L*/gb*-D**	0	3.6	4.4	1	Gel	51	5.0
***tri*-L*/gb*-D(0.5)**	0.5	8.0	0	0.5	Gel	300	8.4
***tri*-L*/gb*-D(1)**	1.0	8.0	0	1	Gel	30	51.6
***tri*-L*/gb*-D(2)**	2.0	8.0	0	2	Gel	<1	105.0
***tri*-L*/gb*-D(5)**	5.0	8.0	0	5	Gel	<1	92.3
***tri*-L*/gb*-L(5)**	5.0	0	10.1	0 (5)	Sol	-	-

^1^ The polymer solutions were mixed at 37 °C. The total graft copolymer concentration was fixed at 8.0 wt%. ^2^ L-Lactide unit (mol)/D-lactide unit (mol). The numbers in parentheses indicate the L-lactide unit in the block copolymer (mol)/L-lactide unit in the graft copolymer (mol).

## Data Availability

All data and materials are available on request from the corresponding author. The data are not publicly available due to ongoing research using a part of the data.
